# Continuous production of *Neisseria meningitidis* outer membrane vesicles

**DOI:** 10.1007/s00253-019-10163-z

**Published:** 2019-11-01

**Authors:** Matthias J.H. Gerritzen, Lilli Stangowez, Bas van de Waterbeemd, Dirk E. Martens, René H. Wijffels, Michiel Stork

**Affiliations:** 1 Institute for Translational Vaccinology (Intravacc), Process Development Bacterial Vaccines, P.O. Box 450, 3720 AL Bilthoven, The Netherlands; 2 Bioprocess Engineering, Wageningen University, P.O. Box 16, 6700 AA Wageningen, The Netherlands; 3 Faculty of Biosciences and Aquaculture, Nord University, P.O. Box 1409, 8049 Bodø, Norway; 4 Dept. Drug Substance Development, Janssen Vaccines and Prevention, Archimedesweg 4-6, 2333 CN Leiden, The Netherlands

**Keywords:** Outer membrane vesicles, OMV, *Neisseria meningitidis*, Continuous processing, Chemostat

## Abstract

Outer membrane vesicles (OMVs) are nanoparticles secreted by Gram-negative bacteria that can be used for diverse biotechnological applications. Interesting applications have been developed, where OMVs are the basis of drug delivery, enzyme carriers, adjuvants, and vaccines. Historically, OMV research has mainly focused on vaccines. Therefore, current OMV production processes have been based on batch processes. The production of OMVs in batch mode is characterized by relatively low yields and high costs. Transition of OMV production processes from batch to continuous processes could increase the volumetric productivity, reduce the production and capital costs, and result in a higher quality product. Here, we study the continuous production of *Neisseria meningitidis* OMVs to improve volumetric productivity. Continuous cultivation of *N*. *meningitidis* resulted in a steady state with similar high OMV concentrations as are reached in current batch processes. The steady state was reproducible and could be maintained for at least 600 h. The volumetric productivity of a continuous culture reached 4.0 × 10^14^ OMVs per liter culture per day, based on a dilution rate of 1/day. The tested characteristics of the OMVs did not change during the experiments showing feasibility of a continuous production process for the production of OMVs for any application.

**Electronic supplementary material:**

The online version of this article (10.1007/s00253-019-10163-z) contains supplementary material, which is available to authorized users.

## Introduction

Outer membrane vesicles (OMVs) are nanoparticles produced by Gram-negative bacteria. OMVs are non-replicative and consist of lipopolysaccharides (LPS), phospholipids, and periplasmic and membrane-bound proteins (Kuehn and Kesty 2005). Biologically, OMVs play roles in intercellular communication, competition, virulence, and nutrient acquisition (Kulp and Kuehn 2010). In biotechnology, OMVs can be used as enzyme carriers (Park et al. 2014), as drug delivery vehicles (Wang et al. 2018), as vaccines (Acevedo et al. 2014), or as adjuvants (Tan et al. 2018).

Most research on OMVs has focused on vaccine development. Especially the development of OMV-based vaccines for serogroup B *Neisseria meningitidis* (Nm) has been researched extensively (Granoff 2010; Panatto et al. 2011). Nm OMVs have been successfully used as prophylactic vaccines to prevent outbreaks of meningococcal disease (Bjune et al. 1991; Cassio de Moraes et al. 1992; Sierra et al. 1991). Moreover, OMVs are included in the currently available serogroup B vaccine Bexsero (Serruto et al. 2012). Traditionally, the production processes of Nm OMV vaccines have been based on the extraction of vesicles from biomass using detergents (dOMV). This was necessary to reduce the endotoxicity of Nm LPS. Genetic detoxification of Nm LPS allowed the use of detergent-free extracted OMVs (eOMVs) as well as spontaneously released OMVs (sOMVs) (van der Ley and van den Dobbelsteen 2011). sOMVs are released by *N*. *meningitidis*, and other bacteria, during normal growth, without the use of any detergents as used for dOMVs or chelating agents as used for extracted OMVs. Compared with both dOMVs and eOMVs, the sOMVs have a different biochemical composition and superior immunogenicity (van de Waterbeemd et al. 2010). However, the low yield of sOMV production processes is a challenge for vaccine production. If the sOMV yield could be improved, sOMVs are the preferred basis for vaccine development, instead of dOMVs or eOMVs. Additionally, sOMVs can be purified with a simplified process as the vesicles can be directly purified from the supernatants of bacterial cultures without any extraction steps. Besides the use as vaccines, high-yield sOMV production processes would allow the use of sOMVs for other biotechnological applications.

Recently, we showed high yields of sOMV from a batch cultivation (Gerritzen et al. 2019b). This was reached by using a production strain with reduced linkage of the outer membrane to the peptidoglycan in combination with sulfur source depletion (Gerritzen et al. 2019a), and high dissolved oxygen levels (Gerritzen et al. 2018). Cysteine depletion triggers OMV release (Gerritzen et al. 2019a; van de Waterbeemd et al. 2013b). However, this mild stress also results in growth arrest and accumulation of undesired components, like DNA that complicates filtration and ammonium that inhibits the nuclease required for DNA removal (van de Waterbeemd et al. 2013b).

Despite the recent developments for improved OMV production processes, higher yields will facilitate the use of OMVs as low-cost vaccines, adjuvant, enzyme carrier, or drug delivery vehicle. Continuous bioprocessing may enable high yield OMV production because it results in higher equipment utilization rates, reduced cycle times, and smaller facility footprints (Pollock et al. 2017), which in turn results in lower production and investment costs. Conversion of batch processes to continuous processes has already shown to be an improvement in many industries (Konstantinov and Cooney 2015). The potential of continuous bioprocessing for the production of biopharmaceuticals shows great potential and has been recognized as a paradigm shift in biologicals production (Konstantinov and Cooney 2015). Additionally, development of continuous biopharmaceutical production has been encouraged by the FDA (Allison et al. 2015; Lee et al. 2015). The aim of this study is to assess the use of steady-state chemostat cultures as an upstream process for continuous processing to obtain a high volumetric productivity of Nm sOMVs. We first assess the production of OMVs in a continuous culture. Then, we further characterize the pre-steady-state phase of the culture. The reproducibility of OMV production was assessed by comparing five replicate steady-state continuous cultures. Optimization of the volumetric productivity was assessed by testing different dilution rates. Lastly, we compare continuous OMV production to the production of OMVs in batch and fed-batch cultures.

## Methods

### Bacterial Strain

A derivative of the H44/76 isolate of *Neisseria meningitidis* serogroup B (NIBSC 2724) (Holten 1979) was used as described previously (Gerritzen et al. 2017). In brief, the strain was non-encapsulated due to a *siaD* knockout (van der Ley et al. 2001) and has reduced LPS-toxicity from an *lpxL1* deletion. This strain has further improved vesicle formation due to the *rmpM* deletion, lacks the major abundant outer membrane protein PorA (Tommassen et al. 1990), and has improved interaction with dendritic cells by *lgtB* deletion (Steeghs et al. 2006).

### Chemostat cultures

Continuous cultures with a working volume of 2 L were performed in 5-L benchtop bioreactors (Applikon) with an H/D ratio of 1.6 based on total volume. The culture medium was chemically defined without animal-derived components containing glucose, amino acids, salts, iron, and trace elements (Baart et al. 2007b). The reactors were controlled using a Tryton^i^ (Pierre Guerin) that controlled the temperature at 35 ± 0.5 °C and pH at pH 7.2 ± 0.05 using 1 M HCl and 1 M NaOH. Dissolved oxygen tension was measured using polarographic oxygen sensors (InPro 6850i, Mettler Toledo) that were calibrated at 100% in air-saturated medium of 35 °C. The cultivations were controlled at 30% air saturation by increasing agitation rate in the batch phase of the cultivation (300–1000 RPM) and mixing of oxygen and air in the headspace aeration (fixed flow rate of 1 L/min). The off-gas composition was measured by a mass-spectrometer (Prima δb, Thermo Scientific). Feed and bleed pumps were started after 8 ± 2 h of growth to initiate a continuous culture. The bioreactor weight, the feed medium weight, and the pH titrant solutions were measured by balances to verify the dilution rate. The dilution rate was set to 0.04 h^−1^ unless indicated otherwise. Culture samples were analyzed for biomass density by measuring the optical density at 590 nm. Steady state was assumed after 3 dilutions based on steady bacterial density measurements and carbon dioxide emission.

### Analytical

Filtered culture samples (0.22 μm pore size) were measured by nanoparticle tracking analysis on a NanoSight NS500 with 488 nm laser module and sCMOS camera (Malloy and Carr 2006). This method was used as a direct method for OMV quantification as the background number of particles in the growth medium are neglectable (Gerritzen et al. 2017). Temperature was controlled at 25 °C and measurements (10 captures of 30 s) were analyzed with the NTA 3.2 software build 3.2.16. Measurements were taken under flow using the automated script described previously (Gerritzen et al. 2017). Residual genomic DNA was determined with a dsDNA assay based on fluorescence. In brief, sterile-filtered culture samples were incubated with Quant-iT PicoGreen dsDNA reagent (Invitrogen), and fluorescence was measured to quantify the DNA concentration based on a calibration curve with salmon sperm DNA standard (Invitrogen).

### Nutrient and metabolite analysis

Amino acids in culture supernatants were measured using the method based on derivatization by orthophtalic anhydride and high-performance liquid chromatography (HPLC) as described in (Dorresteijn et al. 1996; van de Waterbeemd et al. 2010). Organic acids were analyzed by HPLC on a Waters Acquity Class-H (Waters) HPLC system that was equipped with an Acclaim Organic acid guard column (3 × 10 mm, 5 μm, Thermo Scientific) and a Luna® Omega 3 μm polar C18 100 Å 4.6 mm × 150 mm LC column (Phenomenex). The eluent was 0.1 μm sterile-filtered 20 mM potassium phosphate pH 2.5 20 mM, set to pH 2.5 using 4 M HCl. Isocratic elution was performed at 1 mL/min followed by UV (210 nm) and refractive index detection. Samples from bacterial culture supernatants, standards, and controls were first 0.22 μm filtered, to the filtered supernatant (284 μL) concentrated phosphoric acid (10 μL, 14.7 M) containing propionic acid as ISTD was added. Samples were mixed vigorously and subsequently centrifuged 10 min, 15.000×*g* (Thermo Scientific MicroCL 21R) at ambient temperature. Supernatants were collected and analyzed for organic acid content. Organic acid contents were compared with a standard mixture containing l-glutamic acid, d-(-)-tartaric acid, d-(+)-malic acid, l-(+)-lactic acid, citric acid, acetic acid, succinic acid, and fumaric acid of which a calibration curve was constructed. All chromatographic parameters were calculated using the Chromeleon software (v. 7.2, SR 8, Thermo Fisher Scientific). Ammonium was measured in sterile-filtered samples using the BioProfile 100 plus (Nova Biomedical). Glucose concentration was determined by ^1^H-NMR as previously described (Baart et al. 2007a).

## SDS-PAGE

For the protein analysis, OMVs were purified from culture samples by initial removal of biomass by centrifugation at 3000×*g* for 20 min at 4 °C. Next, the supernatant was sterile filtered using a Nalgene™ Rapid-Flow™ filter unit (Thermo Fisher Scientific) containing a PES membrane with a 0.2-μm cutoff. Then, the sterile filtrate pool was concentrated 10–15 times using Amicon Ultra-15 Centrifugal filter units (Merck Millipore) at 4000×*g* for 40 min at room temperature. Finally, the concentrated filtrate was diafiltrated, in the same unit, with 2 volumes of buffer (100 mM Tris-HCl, pH 8.6) by centrifugation (4000×*g*, 15 min, room temperature). Purified OMVs were assessed for total protein content by the Lowry protein assay using Peterson’s modification. OMVs corresponding to 4 μg of protein were loaded on a precast polyacrylamide gel (Lonza) to perform SDS-gel electrophoresis. The electrophoresis was run at 140 V for 90 minwith Accugene 1× Tris-Glycine SDS buffer. To determine the molecular weight, a Pierce^TM^ pre-stained protein weight marker (Thermo Fisher Scientific) was used. The staining of the gel was performed by InstantBlue protein stain (Expedeon) for 1 h.

## Results

### Continuous cultivation of *N*. *meningitidis*

**Fig. 1:**
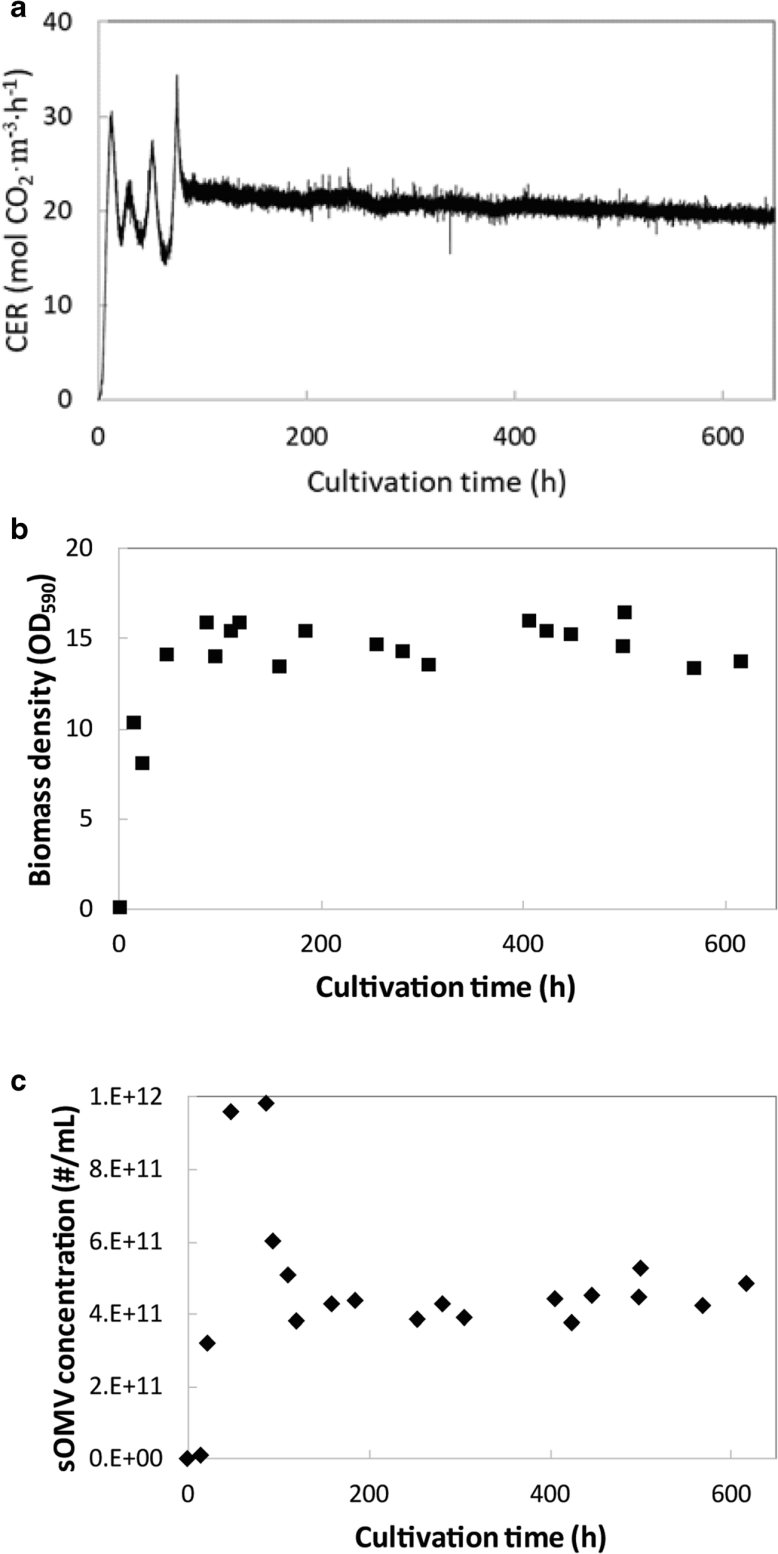
Continuous production of *N*. *meningitidis* OMVs in a chemostat at a dilution rate of 0.04 h^−1^. A steady state of the chemostat culture was reached after 100 h, as shown by the steady carbon dioxide evolution rate (CER) (**a**), biomass density (**b**), and the OMV concentration (**c**). The culture shown is representative for the 5 replicates that were done

A chemostat culture for the continuous production of Nm sOMVs was started as a batch culture. After a short lag-phase (< 1 h), growth was exponential. Dilution of the culture with growth medium was started at a rate of 1/day after 8 h of cultivation. A steady state was reached after 100 h, which equals 4 dilutions of the culture (Fig. 1). This steady state could be maintained for at least 600 h. The steady-state biomass concentration was OD_590nm_ = 14 ± 2. Interestingly, steady-state OMV concentrations reached 4 × 10^11^/mL, which is similar to the maximum sOMV concentration reached in batch cultures in which OMV release was triggered by cysteine depletion (Gerritzen et al. 2018; Gerritzen et al. 2019a).

**Fig. 2.**
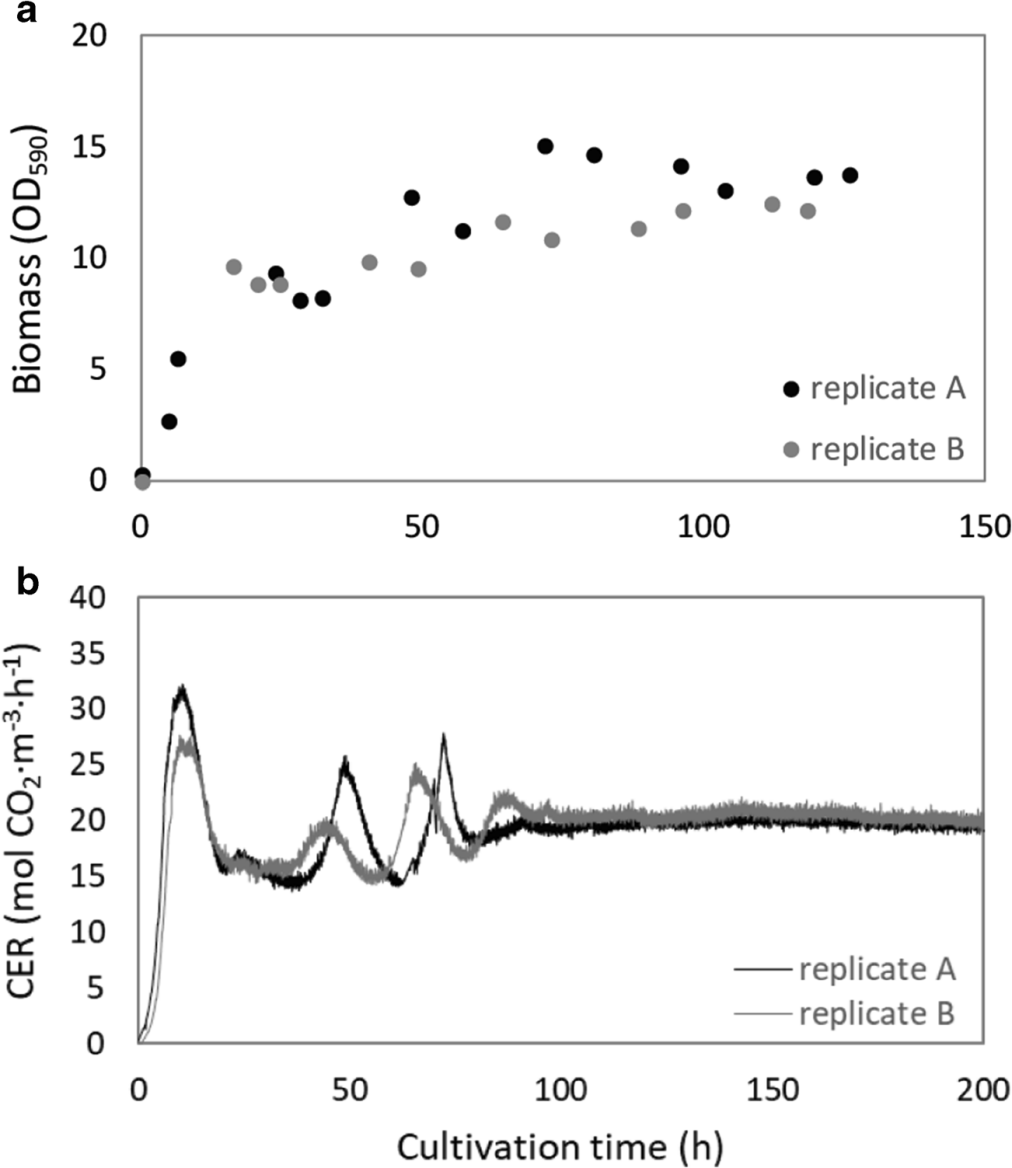
Pre-steady-state of the continuous cultivation. Two replicate chemostat cultures were started to characterize the pre-steady-state phase. **a** Shows the biomass concentration measured by optical density measurements and the carbon dioxide evolution rate (CER) is shown in **b**

The time to reach the steady state of the culture was characterized by periods of faster and slower growth. We hypothesize that this is a result of the complexity of the growth medium that contains glucose, glutamate, arginine, cysteine, and sulfate. The culture was fed a balanced medium composition that has been designed for batch cultivations (Baart et al. 2007a). We assume that the periods of slower and faster growth correlate with certain substrates becoming limiting and an induction period to switch to alternative substrates. To analyze nutrient utilization during the pre-steady-state period, we performed two additional continuous cultures. The cultures reached steady state based on carbon dioxide emission after approximately 4 dilutions (100 h) of the reactor (Fig. 2). The growth of the replicates and the obtained steady state was similar to each other and to the culture shown in Fig. 1. After 8 h of exponential growth, dilution of the culture was started. At this point, cysteine is the first nutrient to be depleted (Fig. S1). In the next phase, sulfate was sufficiently available and presumably used for biosynthesis of cysteine. Additionally, arginine was depleted, after which Nm is capable of arginine biosynthesis from glutamate. Next, the glutamate concentrations decrease below 20 mM (16 h), after which glutamate concentrations stabilized as well as the carbon dioxide emission. From this moment, significant amounts of malic acid, tartaric acid, and acetic acid have accumulated. After 36 h, the carbon dioxide emission increased again and after 50 h of cultivation glucose was depleted. Upon glucose limitation, the residual glutamate was consumed and a steady state was reached. In this steady state, the biomass yield on glucose and on glutamate is respectively 0.42 ± 0.04 g_dw_/g_glucose_ and 0.37 ± 0.05 g_dw_/g_glutamate_. Based on the glucose and glutamate depletion, we assumed that the steady state was limited by the carbon source. To assess this limitation, a steady-state culture was supplemented with a bolus feed of glucose to increase the glucose concentration in the reactor with 20 mM (Fig. S2). In the first hour after addition, a decrease in bacterial density was observed that is caused by the volume of the addition. After this, an increase in bacterial growth was observed by an increasing optical density and increased carbon dioxide emission after this addition, indicating that the steady-state culture is glucose limited.

### Steady-state reproducibility

**Fig. 3.**
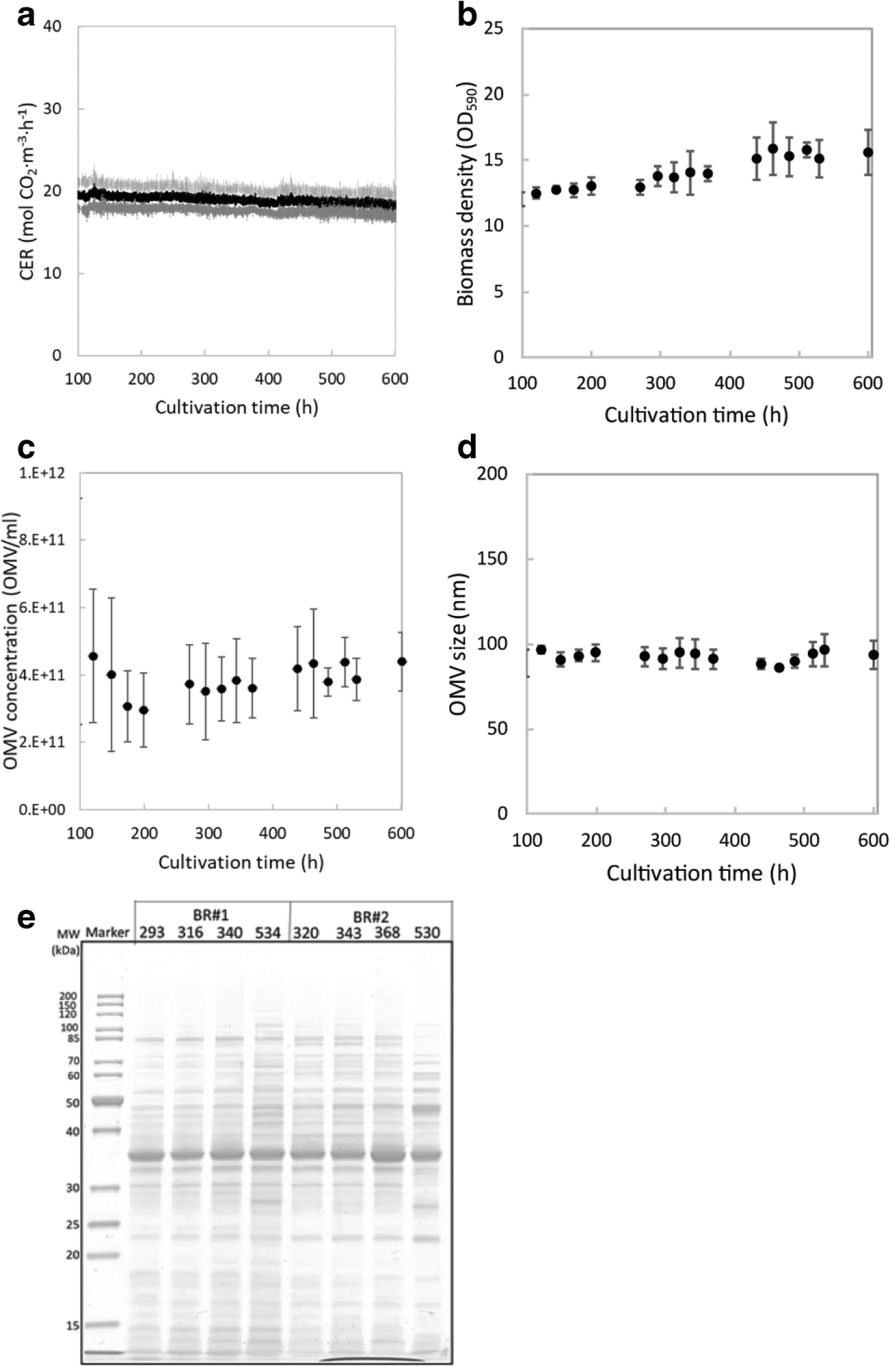
Reproducibility of continuous OMV production in Nm chemostat cultures. Five chemostat cultures were compared to assess the culture to culture variation in OMV productivity. **a** Shows the mean carbon dioxide evolution rate (CER, black line) and standard deviation (grey line). **b** Shows the average biomass density. **c** Shows the OMV concentration and **d** shows the OMV size. Error bars indicate the standard deviation from the mean. The protein composition is shown of OMVs purified from two replicates of a steady-state chemostat culture at different times in the cultivation (**e**)

The reproducibility of the obtained steady state was assessed by comparing 5 replicate cultures (Fig. 3). Culture to culture variability is low as indicated by the standard deviation of the carbon dioxide emission rate. A small decreasing trend can be observed that is caused by a lowered inflow of feed medium due to peristaltic pump tubing wear. Biomass concentrations are reproducible between cultures. A small increasing trend in biomass concentration can be observed that is caused by the altered bioreactor dilution rate because of the changes in the bioreactor volume and medium inflow mentioned earlier. Steady-state OMV concentrations were on average 4 × 10^11^/mL. Steady-state OMV productivity was maintained for at least 500 h (20 dilutions). OMVs were purified from two of the replicates at different time points in the culture and showed similarity based on their protein composition (Fig. 3D).

### Optimization of the continuous sOMV production

**Fig. 4.**
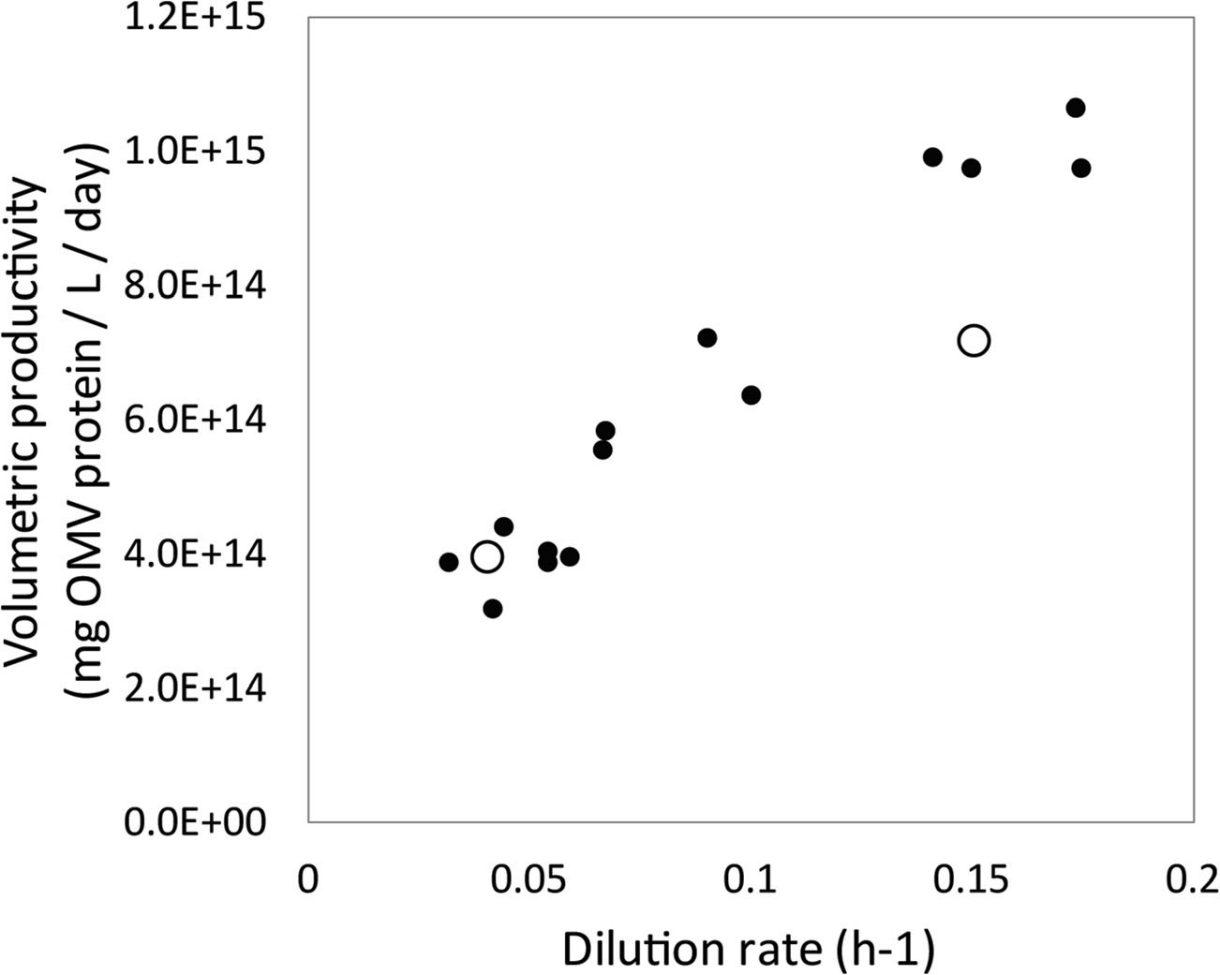
Volumetric OMV productivity as a function of the dilution rate. The volumetric productivity of sOMVs in an accelerostat culture of Nm shows to be linearly related to the dilution rate for growth rates between 0.03 and 0.18 h^−1^ (solid circles) The volumetric productivities of steady-state cultures at 0.04 h^−1^ and 0.15 h^−1^ are depicted as open circles

The dilution rate is a critical process parameter of continuous production processes. The continuous cultures described here so far were based on a practical dilution rate of 1/day (0.04 h^−1^). To optimize the volumetric productivity, an accelerostat culture was performed by slowly increasing the dilution rate (α_D_ = 0.0055 h^−2^) of a steady-state chemostat culture. Previously, using the same accelerostat culture, we already showed that the specific productivity of Nm was only minorly influenced by the growth rate between 0.03 and 0.16 h^−1^ (Gerritzen et al. 2018). Since the specific productivity remains constant, the volumetric productivity increases linearly with the dilution rate (Fig. 4). Thus, operating the continuous culture at 0.15 h^−1^ shows the highest volumetric productivity of 1.0 × 10^15^ OMVs per liter reactor volume per day.

Next, a chemostat was run at 3.6/day (0.15 h^−1^) to confirm the results from the accelerostat culture. For this, the dilution rate of a steady-state culture at 0.04 h^−1^ was increased to 0.15 h^−1^ (data not shown). This steady state at 0.15 h^−1^ showed an OMV concentration of 2.1 × 10^14^ OMVs/L, which was slightly lower than the OMV concentration of 2.5 × 10^14^ OMVs/L in the accelerostat culture at this dilution rate. As a result, the volumetric productivity was 7.4 × 10^14^ OMVs/L/day, which is slightly lower than the expected volumetric productivity of 1.0 × 10^15^ OMVs/L/day based on the accelerostat data. Taken together, a dilution rate of 0.15 h^−1^ shows an increased volumetric productivity of compared with the volumetric productivity of 4.0 × 10^14^ OMV/L/day at a dilution rate of 0.04 h^−1^.

## Comparison to batch and fed-batch production

The production of OMVs in continuous mode was compared with the production in batch and fed-batch mode. We compared these cultures (data shown in Fig. S3) to the continuous production of OMVs (Table 1). For this, we assumed that 20 days of harvest could be obtained from a continuous bioreactor per month, while for the batch process one harvest can be obtained per week. For the fed-batch production mode, we assumed an identical throughput time of 1 week as for the batch process since the fed-batch process requires only 12 h more cultivation time than the batch process. Additionally, we assumed similar recoveries of OMVs in downstream purification regardless of the OMV production method. Based on these assumptions, 16-fold more volume could be processed from a continuous reactor on a yearly basis with a dilution rate of 0.15 h^−1^ (Table 1). This corresponds to a 9-fold increase in OMV production in this continuous mode compared with batch mode. DNA release during the steady state of continuous cultivations was significantly reduced compared with the batch process. During batch and fed-batch cultivations, significant amounts of DNA accumulate caused by lysis of the bacteria (Fig. S3). Furthermore, accumulation of ammonium was observed during the glucose-limited fed-batch cultures, which inhibits enzymatic DNA degradation during OMV purification. Continuous OMV production is thus an improved method over batch and fed-batch production.

**Table 1.** Production characteristics of batch, fed-batch, and continuous OMV production. Benchtop *N. meningitidis* cultures were operated in three different operating modes. Batch mode is characterized by triggered OMV release through cysteine depletion. In fed-batch mode, glucose and glutamate was added to the culture to prolong the OMV releasing state. The continuous culture represents the steady-state values of a culture with a dilution rate of 1/day. Values represent the mean and standard deviation of triplicate cultures, except for the continuous culture at the dilution rate of 3.6/day that represents a single replicate. The first six lines from "cultivation time (h)" to "volumentric productivity" are measured values. The next two " harvest volumes per campaign" and "campaigns per year" are assumptions. The last line "yearly productivity" is calculated.

	Batch	Fed-batch	Continuous	Continuous
Cultivation time (h)	24	40	1 volume/24 h	3.6 volumes/24 h
OMV (10^13^/L)	38 ± 6	52 + 9	40 ± 7	21
Biomass density (OD_590_)	8.7 ± 1.5	11.1 ± 0.3	14 ± 1.6	-
DNA (mg/L)	1.3 ± 0.2	0.48 ± 0.02	0.09 ± 0.03	-
Ammonium (mM)	46 ± 2	51 ± 3	34 ± 5	-
Volumetric productivity (10^14^ OMV/L/Day)	3.8	5.2	4.0	7.4
Harvest volumes per campaign	1	1	20	72
Campaigns/year	52	52	12	12
Yearly productivity (10^13^/L bioreactor volume)	1976	2704	9600	17712

## Discussion

Continuous cultivations of Nm at a dilution rate of 1/day showed high and reproducible OMV concentrations. Increased volumetric productivities could be obtained, compared with batch and fed-batch cultivations. Further intensification of the process is possible by optimizing the volumetric productivity by increasing the dilution rate. Continuous OMV production at a dilution rate of 3.6/day results in a 9-fold increase in OMV production compared with batch-wise OMV production.

The start of the continuous cultivation at a dilution rate of 1/day showed an adjustment period of multiple dilutions, possibly caused by the complexity of the medium. The steady state was characterized by depletion of both carbon sources glucose and glutamate, as well as cysteine and arginine. Addition of glucose to a steady-state culture resulted in bacterial growth, indicating that the culture was glucose limited. Future medium optimization should reduce the complexity of the medium, resulting in a single nutrient limitation that possibly reduces the time required to reach a steady state. Additionally, steady-state biomass productivity can be optimized by future medium optimization. Specific OMV productivities can be further enhanced by inducing OMV release by for example high dissolved oxygen concentrations (Gerritzen et al. 2018).

Dilution rates of continuous production processes can be optimized to reach their maximum volumetric productivity. In this study, increased dilution rates were assessed in an accelerostat culture. At dilution rates above 0.18 h^−1^, the accelerostat culture showed reduced biomass concentrations and was stopped. The maximum specific growth rate of Nm in batch cultures on this medium is 0.5 h^−1^, indicating possibilities for operating continuous processes at higher dilution rates. The fact that higher dilution rates were not feasible in the accelerostat culture is possibly caused by a different nutrient consumption profile at different growth rates. Additionally, the release of OMVs during exponential growth in batch cultures is low (van de Waterbeemd et al. 2013b). Dilution rates of 0.18 h^−1^ and above are thus not expected to further improve the volumetric productivity due to an anticipated decrease in specific productivity. Therefore, the volumetric OMV productivity on this medium composition is maximized at dilution rates just below 0.18 h^−1^. To compare the continuous OMV production with batch and fed-batch OMV production, we used the dilution rate of 0.15 h^−1^ (3.6/day) and assumed this state can be maintained for 20 days. This length should be carefully considered based on future research to the stability of Nm upon prolonged cultivations. The genome of Nm is generally known as variable (Schoen et al. 2009), although Nm subcultured after a production cultures showed to be genetically stable for at least 30 generations during exponential growth (van de Waterbeemd et al. 2013a). Besides monitoring of the genetically stability of the production culture, the OMV product should be characterized more extensively and monitored throughout production cultures. In comparison, perfusion-based monoclonal antibody production systems have been described to be able of production periods of over 60 days (Whitaker et al. 1998). The advantage of perfusion systems is that the growth rate can be kept low resulting in less bacterial generations in time. The stability of the production phase of continuous production processes should be thoroughly assessed to develop a continuous OMV production process for pharmaceutical use. Here, we assessed OMV size by NTA demonstrating that the OMV size was consistent throughout the steady state of continuous cultivations of 600 h. Initial analyses of the OMV protein profile by SDS-PAGE on partially purified OMV samples indicated a similar protein expression pattern, despite few changes in expression level of two samples at 530 and 534 h of cultivation. To obtain a harvest window for production, future research should address the stability of the steady state by quantitative analysis methods in prolonged continuous cultures and assess the activity of the product by immunological assays.

This study showed the continuous upstream production of OMVs. Purification of OMVs should be designed in a continuous manner to obtain a fully continuous OMV production process. Continuous separation of OMVs from the biomass, while maintaining the bacterial culture in a steady state, would be the most straightforward initial separation. Tangential flow microfiltration directly on the continuous reactor could be used. Such an approach would have similarities to high cell density perfusion-based cultivation systems (Clincke et al. 2013; Xu and Chen 2016). Interestingly, a Nm perfusion–based cultivation system has been described (Dehottay et al. 2014). This system enabled the biomass production of 58 g dry weight per liter, although the amount of OMVs secreted in the culture was not described (Dehottay et al. 2014). Further purification of OMVs is required to remove soluble proteins and other contaminants such as DNA and membrane fragments. This can be based on ultracentrifugation or gel filtration (Klimentová and Stulík 2015). While these methods are very effective for batch processes, development of a continuous purification process may benefit from alternative methods. For example, affinity chromatography may provide a simultaneous concentration and purification of OMV. Affinity-based OMV purification has already been shown for *E*. *coli* OMVs expressing a His-tag (Alves et al. 2017). To develop a fully continuous OMV production system, additional studies that design and implement OMV purification is needed. This design should include relevant inline controls like the recently marketed inline MALS detector. This could be used to follow OMV formation and or purification in the downstream process.

The development of new technologies for cleaner and more efficient manufacturing are supported by regulatory authorities (Allison et al. 2015), although currently no continuous vaccine production processes have been described. Important aspects as suitable inline process analytical tools and the possibility of mutations introduced during the continuous production have to be addressed before registration of continuous products will be in sight. Continuous biopharmaceutical production processes have been researched for therapeutic proteins (Karst et al. 2018) and small molecules (Kleinebudde et al. 2017). Recently, two production processes of monoclonal antibodies in a fully continuous manner have been described (Godawat et al. 2015; Karst et al. 2016).

This study shows the potential of continuous production of Nm OMVs to reach high volumetric OMV productivities. The high OMV yields could be beneficial for the production of low-cost biotechnological applications based on OMVs such as enzyme carriers. Future development should focus on a fully continuous purification process. Further research on online product quality analysis methods and batch-to-batch variability could be the basis of future continuous OMV production processes for OMV adjuvants and low-cost price OMV-based vaccines.

## Electronic supplementary material

ESM 1 (PDF 522 kb)
