# Supplementary material for: Continuous production of Neisseria meningitidis outer membrane vesicles
Source: Appl Microbiol Biotechnol. 2019 Nov 1;103(23):9401–10. doi: 10.1007/s00253-019-10163-z (PMC6867985; doi:10.1007/s00253-019-10163-z)
Supplement: Supplementary file 1 — (PDF 522 kb) [file 253_2019_10163_MOESM1_ESM.pdf]

Supplementary data

Journal: Applied Microbiology and Biotechnology

manuscript title: Continuous production of *Neisseria meningitidis* outer membrane vesicles

Author names:

Matthias J.H. Gerritzen [1,2]

Lilli Stangowez[1]

Bas van de Waterbeemd[1*]

Dirk E. Martens [2]

René H. Wijffels [2,3]

Michiel Stork [1 **]

Author affiliations

[1] Institute for Translational Vaccinology (Intravacc), Process Development Bacterial Vaccines, P.O. Box 450, 3720 AL Bilthoven, The Netherlands

[2] Wageningen University, Bioprocess Engineering, P.O. Box 16, 6700 AA Wageningen, The Netherlands

[3] Nord University, Faculty of Biosciences and Aquaculture, P.O. Box 1409, 8049 Bodø, Norway

[*]Current address: Janssen Vaccines and Prevention (dept. Drug Substance Development), Archimedesweg 4-6, 2333 CN Leiden, The Netherlands

[**] Correspondence: michiel.stork@intravacc.nl, +31 30 7920 525

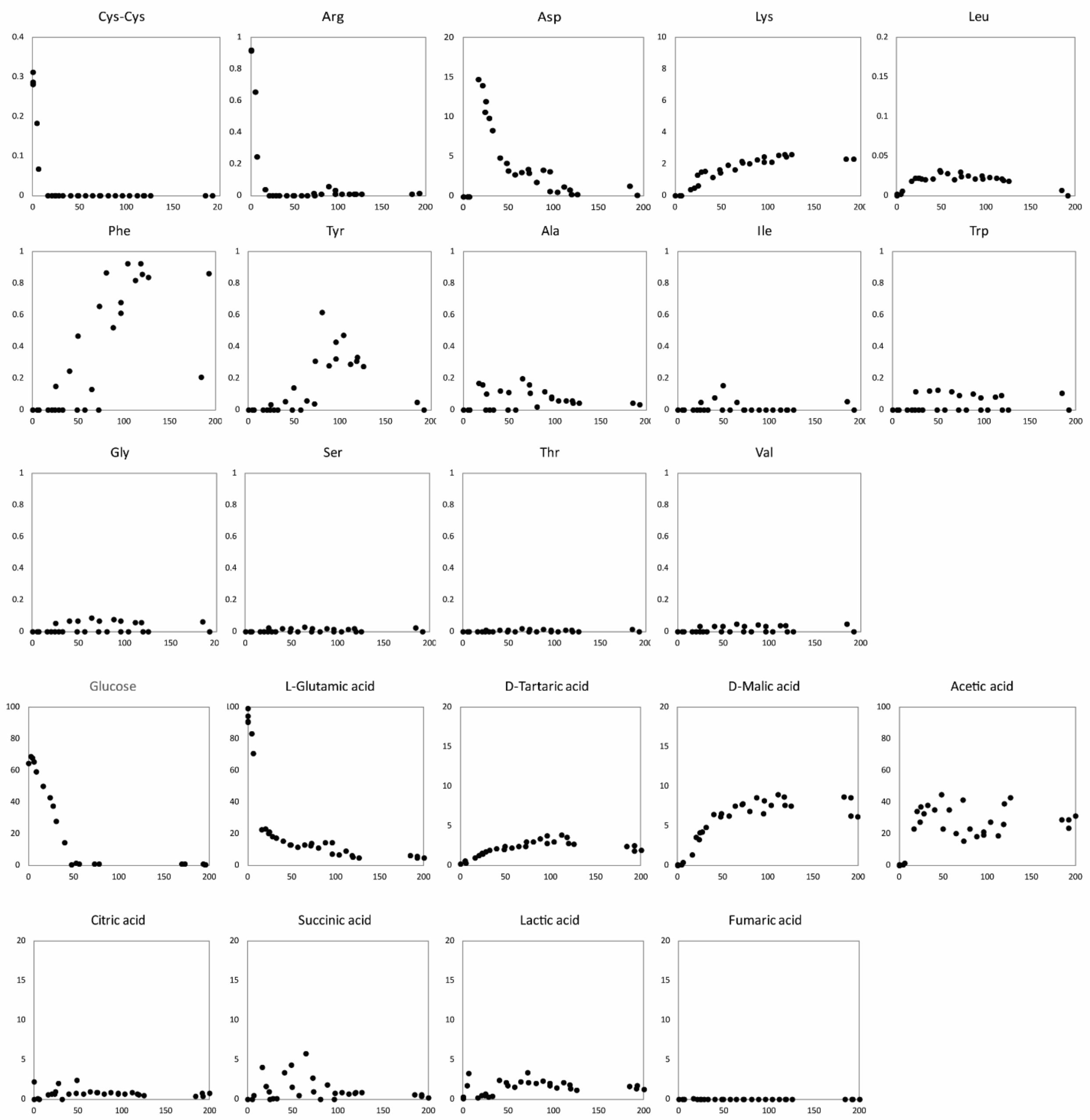

Figure S1. Nutrient and metabolite profile of the chemostat. Measurements represent the overlay of samples of the adaptation phased from two replicate chemostat cultures of Nm with a dilution rate of 1/day. All concentration measurement are in mM. Amino acids cystine (Cys-cys), arginine (arg), aspartate (Asp), lysine (Lys), leucine (Leu), phenylalanine (Phe), tyrosine (Tyr), alanine (Ala), isoleucine (Ile), tryptophan (Trp), glycine (Gly), serine (Ser), threonine (Thr), and valine (Val) are measured, asparagine, glutamine, histidine, methionine, hydroxyproline, and proline were not detected (data not shown).

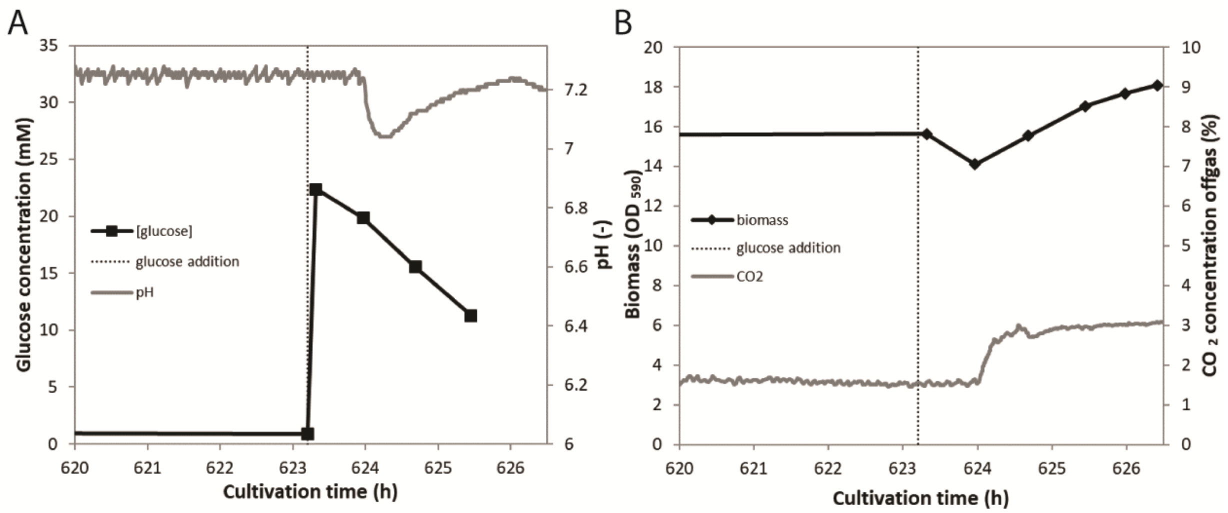

Figure S2. Identification of the limiting nutrient. To a steady state culture of *N. meningitidis*, a shot of glucose solution was added to increase the glucose concentration with 20 mM (graph A, dashed line). The biomass concentration is measured by optical density measurements and the carbon dioxide concentration was measured in the offgas (graph B).

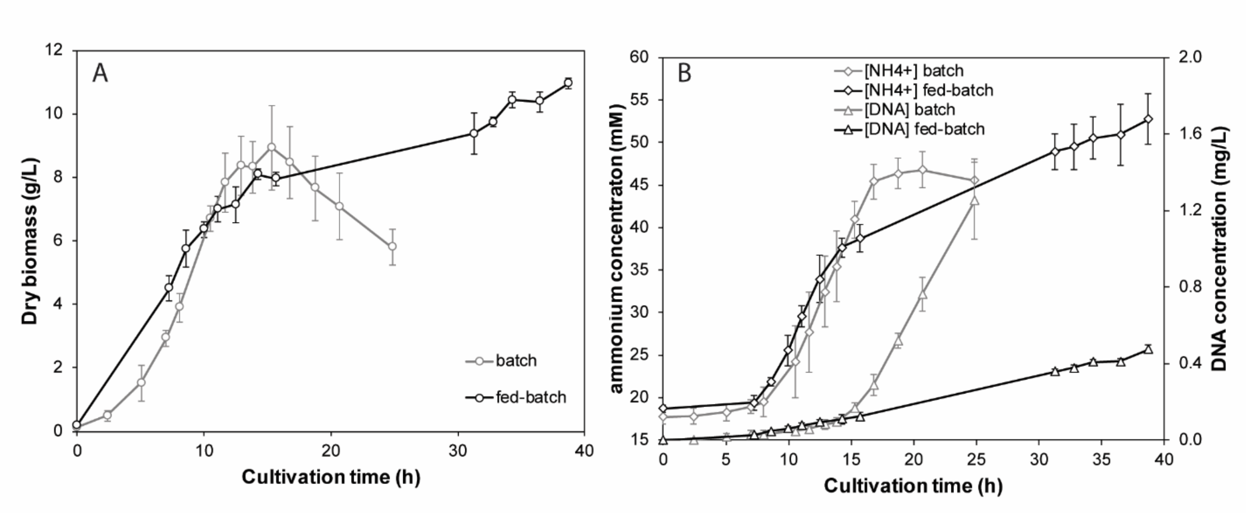

Figure S3. Batch and fed-batch *N. meningitidis* cultures. Nm cultures were operated in batch-mode, as well as in fed-batch mode using a glucose feed solution (Graph A). Accumulation of ammonia and DNA was measured in both cultures (Graph B). Data represents mean value of triplicate cultures; error bars indicate the standard deviation.
